# Foreword to the Focus Issue: science and technology of element-strategic permanent magnets

**DOI:** 10.1080/14686996.2022.2027641

**Published:** 2022-01-28

**Authors:** Satoshi Hirosawa, Kazuhiro Hono

**Affiliations:** Elements Strategy Initiative Center for Magnetic Materials, National Institute for Materials Science, Tsukuba, Japan

Permanent magnet is one of the most important industrial materials in modern society and its function is to generate magnetic flux in a space without any supply of energy. The most powerful and commercially important permanent magnet today is the Nd-Fe-B magnet that appeared in 1983. This material is composed of an iron-based hard magnetic compound, Nd_2_Fe_14_B, as the main constituent, and hence the commercialization of the Nd-Fe-B magnets solved the issue associated with the criticality of Co that was used in the previous Sm-Co based champion magnets. Since Nd is the most abundant lanthanide element with a magnetic moment, the Nd-Fe-B magnet was considered as the final solution in the permanent magnet industry. However, this material had a poor high-temperature performance, particularly low coercivity. To deal with this weakness, heavy rare earth elements (HREE) such as Tb and Dy have been used to improve the coercivity, which raised another issue arising from the criticality of HREE. This raised worldwide attention about a decade ago, and the development of advanced permanent magnets with less critical metals have become active all over the world. As a result, new knowledge and technologies concerning the material science of the high-performance rare earth magnets have been acquired in the last decade.

The science and technology of permanent magnets contain a wide spectrum of disciplines from physics of magnetism, phase equilibria and process sciences. The functionality of a permanent magnet comes from the large hysteresis of magnetization, which is a typical structure-dependent phenomenon. Permanent magnets are normally used in the second quadrant of the hysteresis below the external field called ‘coercivity’ at which magnetization reversal occurs. So, the major issue in the development of permanent magnets has been how to achieve high coercivity by controlling the microstructure of the alloys that contain a hard magnetic compound as a major constituent phase. Another important activity in permanent magnetism is the search for new hard magnetic compounds whose intrinsic hard magnetic properties are comparable or superior to those of Nd_2_Fe_14_B. A blind search with only human experiences over numerous compositional and processing parameters has been proved to be insufficient after decades of research.

Today, the science and technology of permanent magnet materials is in a totally different stage as compared to the situation a decade ago. The first-principles calculations of the ground state magnetism of rare earth magnetic compounds are now possible with great precision and finite-temperature magnetic behaviors are now handled considering the contributions of phonons in the electronic calculations on one hand and by using effective atomistic spin models in mesoscopic scales combined with micromagnetic stochastic simulations or thermodynamic Monte Carlo simulations on the other. The fundamental understandings of microstructures and local magnetic properties have been completely renewed in the case of the Nd-Fe-B magnet by the use of advanced analytical technologies for atomic-resolution structural, chemical, and magnetic properties.

The selection of authors in this Focus Issue is biased to Japanese institutes. Instead of trying to take an international balance, this Focus Issue attempted to update the activities in two decade-long national projects in Japan, namely, Elements Strategy Initiative Center for Magnetic Materials (ESICMM) under the subsidy of the Ministry of Education, Culture, Sports, Science and Technology (MEXT), and the Technology Research Association of Magnetic Materials for High-Efficiency Motors (MagHEM) under the subsidy of the Ministry of Economy, Trade and Industry (METI). The majority of contributions to this Focus Issue are from ESICMM, which has been given the role of laying out the scientific bases of permanent magnets rather than industrial developments.

The paper by Li et al. from NIMS addresses the most frequently asked questions about the coercivities of Nd-Fe-B permanent magnets by summarizing the advances in the understanding of microstructure–coercivity relationships of Nd-Fe-B based permanent magnets. The paper by Koyama and Tsukada from Nagoya University and Abe from NIMS describe the recent understandings of the thermodynamics of formation of the thin intergranular phase, which is necessary to achieve a high coercivity in Nd-Fe-B magnets. The paper by Takahashi, Sepehri-Amin and Ohkubo from NIMS reviews the development of the Fe-rich ThMn_12_-type rare earth magnets which are attracting attention due to their intrinsic magnetic properties superior to those of Nd_2_Fe_14_B. The paper by Trinh et al. from Kyoto University reviews the synthesis of mesoscopic particles of rare-earth permanent magnet compounds via a chemical route from nano-sized precursors. One area that has shown marked advances in the past decade is the theoretical understandings of coercivity on the basis of atomistic spin models of the rare earth permanent magnets constructed from information obtained by the first-principles calculations. Ghoda from Tokyo Institute of Technology summarizes the recent advances in the large-scale first-principles computation of atomistic structure and magnetic properties of near grain-boundary interfaces in Nd-Fe-B and SmFe_12_-SmCu systems. The paper by Tsuchiura et al. from Tohoku University reviews finite temperature magnetic properties in equilibrium and the switching field of the magnetization of particles, based on an atomistic spin model. The paper by Miyashita et al. from The University of Tokyo describes advances in the theoretical computation of finite-temperature dynamics of magnetization process and thermodynamics associated with coercivity phenomena in the spin model. The paper by Okamoto from Tohoku University summarizes the advances in experimental approaches for micromagnetic measurements and analysis of the coercivity mechanism. The paper by Miyake et al. from AIST is a review of the high-precision first-principles calculation of the stability and magnetic properties of the rare earth permanent magnet compounds. The paper by Abe et al. is a brief summary of combined studies of equilibria experiments and theoretical calculations for the construction of a prototype database for thermodynamic assessments using the Calphad-type methods to describe important features of production processes of the multi-component Nd-Fe-B-type permanent magnets.

The invited paper by Hioki from Daido Steel Co. Ltd. provides an account of the development of hot-deformed Nd-Fe-B magnets that pioneered installment of the Dy-free Nd-Fe-B magnet in hybrid electric vehicles in 2016. The paper by Horikawa et al. of Aichi Steel Corporation is a review of recent advancements of anisotropic bonded magnets based on Nd-Fe-B and Sm-Fe-N partly conducted under MagHEM and ESICMM. Another paper by Takagi et al. from AIST reports recent results of their activities on powder materials processing for Sm_2_Fe_17_N_3_ and related materials.

As guest editors, we are confident that this Focus Issue provides a concise summary of the advancements in the science and technology of permanent magnets in Japan during the past decade and perspectives of the future direction of R&D in this field of research.

